# Genetic and Biochemical Evidence That *Enterococcus faecalis* Gr17 Produces a Novel and Sec-Dependent Bacteriocin, Enterocin Gr17

**DOI:** 10.3389/fmicb.2019.01806

**Published:** 2019-08-13

**Authors:** Guorong Liu, Yao Wang, Xue Li, Xu Hao, Duoxia Xu, Yingning Zhou, Arshad Mehmood, Chengtao Wang

**Affiliations:** ^1^Beijing Advanced Innovation Center for Food Nutrition and Human Health, Beijing Engineering and Technology Research Center of Food Additives, Beijing Technology and Business University, Beijing, China; ^2^Beijing Academy of Agricultural and Forestry Sciences, Beijing, China

**Keywords:** *Enterococcus faecalis*, enterocin Gr17, complete genome sequence, gene cluster, antibacterial activity

## Abstract

Bacteriocins are ribosomally synthesized antibacterial peptides or proteins from microorganisms. We report a novel bacteriocin producing strain, *Enterococcus faecalis* Gr17, that was isolated from the Chinese traditional low-salt fermented whole fish product Suan yu. *E. faecalis* Gr17 displayed potent antibacterial activity against foodborne pathogenic and spoilage bacteria. The complete genome of *E. faecalis* Gr17 contained one circular chromosome and plasmid. The gene cluster of a novel bacteriocin designated enterocin Gr17 was identified. The enterocin Gr17 structural gene encodes a precursor of the bacteriocin. Two other transporter genes and an immunity gene within two divergent operons were identified as being associated with enterocin Gr17 secretion and protection. The novel enterocin Gr17 was purified by ammonium sulfate precipitation, cation exchange, gel filtration, and reverse-phase high-performance liquid chromatography. The molecular weight of enterocin Gr17 was 4,531.01 Da as determined by matrix-assisted laser desorption/ionization time-of-flight mass spectrometry and its mature amino acid sequence of enterocin Gr17 was RSYGNGVYCNNSKCWVNWGEAKENIIGIVISGWATGLAGMGR. Sequence alignment revealed that enterocin Gr17 is a class IIa bacteriocin with similarities to enterocin P. The merits of bactericidal activity, sensitivity to enzymes, and pronounced stability to chemicals, temperature (60°C, 30 min and 121°C, 15 min), and pH (2–10) indicated practicality and safety of enterocin Gr17 in the food industry. The complete genome information of *E. faecalis* Gr17 will improve the understanding of the biosynthetic mechanism of enterocin Gr17, which has potential value as a food biopreservative.

## Introduction

Bacteriocins are ribosomally synthesized proteins and protein complexes with a broad spectrum of antibacterial activities against many food-borne pathogens and closely related species (Cleveland et al., 2001). They do not affect cells that produce immune-related proteins (Diep et al., 2007). Bacteriocins from lactic acid bacteria (LAB) are non-toxic, highly potent, and safe, and so have been widely used as preservatives for foods that include vegetables, meats, and other food products (Gálvez et al., 2007; Yang et al., 2014). Bacteriocins may be developed as viable alternatives to antibiotics (Cotter et al., 2013).

*Enterococcus* is a genus of LAB. The bacteria are Gram-positive, catalase-negative, facultative anaerobic, and non-spore forming (Moraes et al., 2013). Enterococci, the first LAB colonizing the infant gastrointestinal tract (GIT) (Fanaro et al., 2010), are also ubiquitous in fermented foods and the environment (Franz et al., 2003; Foulquie Moreno et al., 2006). Furthermore, some *Enterococcus* spp. are commercially available and prevent numerous diseases. As examples, *Enterococcus faecium* SF68^®^ has been used as a food biopreservatives and in the treatment of diarrhea (Kathrani et al., 2016; Holzapfel et al., 2018), *Enterococcus faecalis* Symbioflor 1 is efficacious for the treatment of sinusitis and bronchitis (Habermann et al., 2002), and *E. faecium* JWS 833 enhances cytokine production by dendritic cells (Choi et al., 2012). Thus, enterococci are important for the health of humans and animals, as well as in the food industry and the environment.

Lactic acid bacteria produce antimicrobial substances that include organic acids, hydrogen peroxide, and bacteriocins. Bacteriocins have potent inhibitory activity against sensitive strains of bacteria. There are four classes (I–IV) of bacteriocins (Rea et al., 2011; Kumariya et al., 2019). Class I bacteriocins are <5 kDa. They are posttranslationally modified peptides, which contain non-standard amino acids, such as lanthionine and β-methyllanthionine. The class I bacteriocins comprise three subgroups: class Ia (lantibiotics), class Ib (labyrinthopeptins), and class Ic (sanctibiotics) (Parada et al., 2007). Class II bacteriocins (5–10 kDa) are heat-stable unmodified peptides, which comprise four subgroups: class IIa (pediocin-like bacteriocins), class IIb (two-peptide bacteriocins), class IIc (circular bacteriocins), and class IId (linear and non-pediocin-like bacteriocins) (Cui et al., 2012). Class III bacteriocins (>30 kDa) are heat-labile proteins, which include colicins, helveticin M, helveticin J, and enterolysin A (Kumariya et al., 2019). Class IV bacteriocins are large complexes with lipid or carbohydrate moieties. They are now termed bacteriolysins (Liu et al., 2014). Generally, class IIa bacteriocins consist of a conserved YGNGV motif and disulfide bond linkage (Perez et al., 2014).

Enterocin is a bacteriocin obtained from the *Enterococcus* species. Numerous enterocin-producing enterococci and enterocins have been reported. They include enterocin A from *E. faecium* CTC492 (Aymerich et al., 1996), enterocin B from *E. faecium* T136 (Casaus et al., 1997), enterocin P from *E. faecium* P13 (Cintas et al., 1997), and enterocin Q from *E. faecium* L50 (Cintas et al., 2000). Class IIa enterocins are synthesized as a precursor with an N-terminal signal peptide, which is cleaved by adenosine triphosphate (ATP)-binding cassette (ABC) transporter (Havarstein et al., 1995) or the Sec secretion system (Cintas et al., 1997). The type of N-terminal signal peptide determines the synthetic mechanism of enterocin. Although bacteriocins and producer cells play an essential role in the food industry, few studies have examined the biosynthetic mechanism and practical application of enterocins.

Virulence factors and antibiotic resistance are important in the pathogenicity of *E. faecalis*. Many virulence genes have been reported in enterococci, including cytolysins (*cylA*, *cylB*, and *cylM*), gelatinase (*gelE*), sex pheromones (*cpd*, *cob*, and *ccf*), aggregation substance gene (*agg*), and extracellular surface protein gene (*esp*, *efaAfs*, and *efaAfm*) (Barbosa et al., 2010; Chajęcka-Wierzchowska et al., 2016). Moreover, several antibiotic resistance genes have been described in enterococci. These include genes conferring resistance to erythromycin (*ermB*, *ermC*), tetracycline (*tetM*, *tetS*, *tetO*, *tetK*, and *tetL*), ciprofloxacin (*gyrA* and *parC*), ampicillin (*bla*), and vancomycin (*vanA*, *vanB*, and *vanC*) (Ohmomo et al., 2000; Gevers et al., 2003; Comunian et al., 2010; Guo et al., 2017).

In the present study, the complete genome sequence of *E. faecalis* Gr17, a novel strain isolated from a Chinese traditional low-salt fermented whole-fish product, was determined. The biosynthetic mechanism of enterocin Gr17, a novel bacteriocin from *E. faecalis* Gr17, was analyzed by bioinformatic analyses. Furthermore, the physicochemical characterization and antibacterial activity of purified enterocin Gr17 were determined. The genome information of *E. faecalis* Gr17 and the antibacterial properties of enterocin Gr17 provide the theoretical foundation for the potential use of the bacteriocin as a food preservative.

## Materials and Methods

### Samples and Bacterial Culture Conditions

Samples of the Chinese traditional low-salt fermented whole-fish product known as Suan yu were collected from the Dong ethnic minority regions in Liping, Guizhou Province, China. All LAB strains were cultured in MRS medium without agitation at 37°C. The details of each medium used for the indicator strain are provided in Table 1. All bacteria were grown at 37°C and stored at −80°C in culture broth containing 20% glycerol (v/v).

**TABLE 1:** Antimicrobial spectrum of enterocin Gr17.

**Indictor strains**	**Source^a^**	**Media**	**Sensitivity^b^**
*Listeria monocytogenes* 35152 (4b)	ATCC	TSYEB	+++
*L. monocytogenes* 54002 (1/2a)	CMCC	TSYEB	+++
*L. monocytogenes* 19112 (2a)	ATCC	TSYEB	++
*L. monocytogenes* 19113 (3a)	ATCC	TSYEB	+++
*L. monocytogenes* 19114 (4a)	ATCC	TSYEB	++
*L. ivanovii* 19119	ATCC	TSYEB	++
*L. grayi* 19120	ATCC	TSYEB	++
*L. seeligeri* 35967	ATCC	TSYEB	++
*L. innocua* 33090	ATCC	TSYEB	+++
*Staphylococcus aureus* 6538	ATCC	TSB	++
*S. aureus* 1.128	CGMCC	TSB	+++
*S. aureus* 1.1477	CGMCC	TSB	++
*S. aureus* 26112	CVCC	TSB	+++
*S. aureus* 26923	CVCC	TSB	+++
*S. aureus* 29213	ATCC	TSB	+++
*Bacillus subtilis 10275*	CICC	LB	++
*B. subtilis 1.399*	CGMCC	LB	+++
*B. subtilis 6633*	ATCC	LB	++
*B. subtilis 1.7740*	CGMCC	LB	+++
*B. subtilis 1.15792*	CGMCC	LB	++
*B. subtilis 1.8715*	CGMCC	LB	+++
*B. cereus 63303*	CMCC	LB	+++
*B. cereus 14579*	ATCC	LB	+++
*B. cereus 11778*	ATCC	LB	+++
*B. cereus 1.229*	CGMCC	LB	++
*B. cereus 1.0559*	CGMCC	LB	++
*B. cereus 1.1626*	CGMCC	LB	++
*B. anthracis*	LAB	LB	++
*Brochothrix thermosphacta*	LAB	LB	++
*E. coli* 8739	ATCC	LB	+++
*E. coli* 1.90	CGMCC	LB	+++
*E. coli* 25922	ATCC	LB	+
*E. coli* 10389	CICC	LB	++
*Salmonella* C500	CVCC	LB	+
*Salmonella enteritidis* 13076	ATCC	LB	−
*S. enteritidis* 21513	CICC	LB	+
*S. infantis* 21482	CICC	LB	−
*Enterococcus faecalis* 29122	ATCC	TSB	+++
*Pseudomonas aeruginosa* 21636	CICC	LB	+
*P. aeruginosa* 9027	ATCC	LB	+
*P. fluorescens* 1.55	CGMCC	LB	+
*P. fluorescens* 1.1802	CGMCC	LB	+
*Cronobacer sakazakii* 51329	ATCC	LB	+
*Aspergillus flavus* 40375	CICC	YPD	−
*Penicillium citrinum* 4010	CICC	YPD	−
*Aspergillus niger* 98003	CMCC	YPD	−
*Candida albicans* 98001	CMCC	YPD	+

*^*a*^CMCC, National Center for Medical Culture Collections; ATCC, American Type Culture Collection; CGMCC, China Center of General Microbial Culture Collection; CVCC, China Center of Veterinary Culture Collection; CICC, China Center of Industrial Culture Collection. ^*b*^Inhibition zone (mm): +++, >21 mm; ++, 11–20 mm; +, 1–10 mm; −, no inhibition.*

### Isolation of Bacteriocin-Producing LAB

Liquid fermented fish samples (three cans) were mixed with 80 mL of sterile 0.9% NaCl. Serial dilutions were made using sterile 0.9% NaCl, and 1 mL of each dilution was spread on MRS agar. The plates were cultured at 37°C for 24 h. The 589 bacterial colonies that developed were each cultured in 2 mL of MRS broth for 24 h at 37°C. Each culture was centrifuged at 8,000 × *g* for 20 min at 4°C. The supernatant was recovered from each culture, the pH was adjusted to 7.0, and it was filtered through a 0.22-μm filter. The antibacterial activity in the filtered supernatant was determined by the well diffusion method (Voulgari et al., 2010). The 22 selected strains that displayed more pronounced antibacterial activity against the indicator strains (*Listeria monocytogenes* and *Escherichia coli*) were further tested with other indicator strains (*Staphylococcus aureus*, *Bacillus subtilis*, and *Bacillus cereus*). *E. faecalis* Gr17 displayed pronounced antibacterial activity and was selected for the subsequent experiments.

### DNA Purification and Identification of Bacteriocin From *E. faecalis* Gr17

Strain Gr17 was cultured in MRS medium at 37°C without agitation. Genomic DNA was purified using the QIAamp DNA Mini Kit (Qiagen, Germany) according to instructions provided by the manufacturer. The concentration and purity of genomic DNA were assessed using a NanoDrop 2500 spectrophotometer (Thermo Fisher Scientific, MA, United States). Genotypic identification was performed according to the 16S rRNA gene sequence. The extracted genomic DNA was used as the PCR template. The primers were as follows: 16S rRNA-forward, 5′-AGAGTTTGATCCTGGCTCAG-3′; 16S rRNA-reverse: 5′-GGTTACCTTGTTACGACTT-3′. The 16S rRNA amplified as previously described (Rushdy and Gomaa, 2013) was sequenced by Sangon Biotech (Shanghai, China), after which the sequence was used for a BLAST search of the GenBank database.

### Genome Sequencing and Assembly

The complete genome of strain Gr17 was prepared using the PacBio platform. According to the protocol, a 20-kb DNA library was constructed and sequenced with Single Molecule, Real-Time (SMRT) technology. The sequences of SMRTCell were assembled by SMRT Pipe version 2.1.1. The reads were *de novo* assembled and polished using the Hierarchical Genome Assembly Process version 3/Quiver.

### Genome Annotation

Gene prediction and annotation were carried out using the Glimmer 3.02 software^1^ and the Rapid Annotation Search Tool (RAST) (Aziz et al., 2008). Additional gene and function protein identification was performed using the Kyoto Encyclopedia of Genes and Genomes (KEGG) database (Kanehisa et al., 2016) and Clusters of Orthologous Groups (COG) of proteins database (Tatusov et al., 2003).

### Purification of Enterocin

Strain Gr17 was grown in 100 mL of MRS broth to an optical density at 600 nm (OD_600_) of 0.4. A defined portion of the culture (0.5% v/v) was used to inoculate 2 L of MRS broth, which was incubated without agitation for 24 h at 37°C. The bacteria were removed by centrifugation at 8,000 × *g* for 20 min at 4°C. The supernatant was precipitated using 70% ammonium sulfate at 4°C and desalted by dialysis in sodium phosphate buffer (pH 6.5) with a cellulose semipermeable membrane (molecular weight cutoff, 1,000). The antibacterial activity of crude extracts was assayed, and the antibacterial samples were stored at −80°C.

The active extracts were further purified using the ÄKTA^TM^ pure system (GE, MA, United States). A SP-sepharose fast flow cation exchange column (16 × 25 mm) was equilibrated with 20 mM sodium phosphate buffer (pH 5.5), and the samples that had been filtered through a 0.22-μm filter were loaded onto the column and eluted with linear gradient from 0 to 1 M NaCl at a flow rate of 1 mL/min. The fractions were collected according to ultraviolet (UV) absorbance, and the antibacterial activity of collected fractions was assayed.

A Sephadex G25 column (26 × 100 mm) was equilibrated with 20 mM phosphate buffer (pH 5.5) and 2 mL of bacteriocin obtained from the cation exchange column was eluted by elution buffer (20 mM phosphate buffer) at a flow rate of 0.5 mL/min. The antibacterial activity of the collected fractions according to UV absorbance was assayed.

A C_18_ reverse-phase column (4.6 × 250 mm, 5 μm; Agilent, CA, United States) equipped with a reversed-phase high-performance liquid chromatography (RP-HPLC) system (Agilent) was used for further purification of bacteriocin. A linear gradient elution with 95% water–acetonitrile (5–95%) containing 0.1% trifluoroacetic acid (TFA) was used as the elution phase. The flow rate was 0.5 mL/min, and absorbance was monitored at 280 nm. The purified bacteriocin, which was designated enterocin Gr17, was collected and used to assess its antibacterial activity.

The antibacterial activity was assayed by the agar well diffusion method. The purity was assayed by tricine–sodium dodecyl sulfate (SDS)–polyacrylamide gel electrophoresis (PAGE). The concentration was determined using a Bicinchoninic Acid Kit (Thermo Fisher Scientific, MA, United States) according to the manufacturer’s instructions.

### Molecular Mass of Enterocin Gr17

The molecular mass of purified enterocin Gr17 was determined by AB SCIEX 4700 matrix-assisted laser desorption/ionization time-of-flight mass spectrometry (MALDI–TOF–MS) in linear mode (Applied Biosystems, CA, United States). Enterocin Gr17 was spotted on a target plate and left to dry. The dried enterocin was mixed with 0.5 μL of matrix solution containing α-cyano-4-hydroxycinnamic acid dissolved in 0.1% TFA (v/v) and 50% acetonitrile (v/v). Spectrometry was performed in positive ion mode.

### Antibacterial Spectrum of Enterocin Gr17

The purified enterocin Gr17 obtained by RP-HPLC was used to determine its antibacterial spectrum against indicator strains containing food spoilage bacteria and food-borne pathogens.

### Sensitivity to Heat, pH, Surfactants, and Proteolytic Enzymes

The purified enterocin Gr17 obtained by RP-HPLC was assessed. To determine the effect of temperature on antibacterial activity, enterocin Gr17 was incubated at 60, 80, and 100°C for 30 min, and at 121°C for 15 min. The residual antibacterial activity was tested, and the sample at 37°C was used as the control.

The pH stability of enterocin Gr17 was estimated by adjusting the pH between 2 and 11 with 1 M NaOH or 1 M HCl. After incubation for 3 h at 37°C, the pH was neutralized to pH 6.5 and the residual antibacterial activity was tested.

The effect of ethylenediaminetetraacetic acid (EDTA), SDS, Tween 20, Tween 80, and urea (1%, v/v, final concentration) on enterocin Gr17 was assessed. After incubation for 3 h at 37°C, the residual antibacterial activity was tested.

The sensitivity of enterocin Gr17 to various proteolytic enzymes was determined by mixing 80 μL of enterocin Gr17 with 20 μL of enzymes (1 mg/mL, Sigma–Aldrich, MO, United States) including pepsin (pH 3.0), papain (pH 6.5), proteinase K (pH 7.5), trypsin (pH 7.6), and chymotrypsin (pH 7.8) for 3 h at 37°C. The control lacked enzyme.

### Statistical Analyses

All experiments were performed in triplicate. Results were analyzed by analysis of variance (ANOVA) and Duncan’s test with the SPSS 23.0 software (SPSS, IL, United States). All results are presented as mean ± standard deviations (SDs). A *P*-value < 0.05 was considered statistically significant.

## Results

### Isolation of Bacteriocin-Producing Strains

A total of 589 single bacterial colonies were isolated from Suan yu. The cell-free supernatant of 22 strains (pH 7.0) showed higher antibacterial activity against indicator strains (*L. monocytogenes* and *E. coli*). Strain Gr17 possessed antibacterial activity against *S. aureus*, *B. subtilis*, and *B. cereus.*

### Identification and Genome Features of Strain Gr17

**FIGURE 1:**
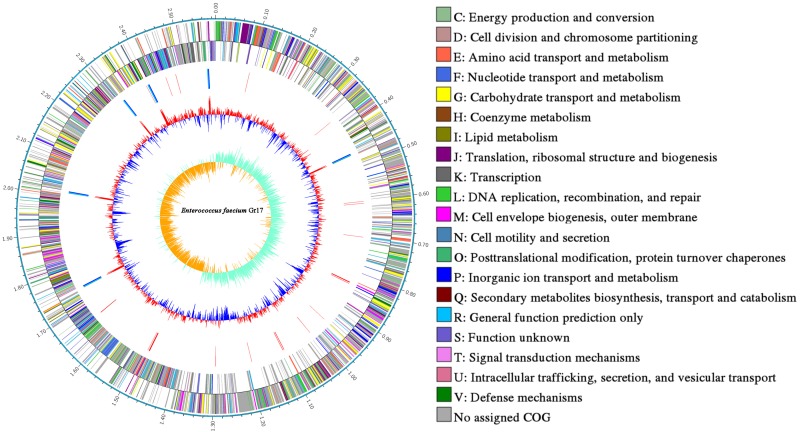
Circular genome map of *Enterococcus faecalis* Gr17. Ring 1: genome sequences. Rings 2 and 3: COG annotated coding sequences. Ring 4: KEGG enzymes. Ring 5: RNA genes. Ring 6: GC content. Ring 7: GC skew. Very short features were enlarged to enhance visibility. Clustered genes, such as several rRNA genes, may appear as one line due to space limitations. The image was created by using Circos software.

The complete genome and 16S rRNA analysis information identified strain Gr17 as *E. faecalis*. The strain was designated *E. faecalis* Gr17. The complete genome of *E*. *faecalis* Gr17 consists of a 2,588,149-base pair (bp) circular chromosome and a 49,643-bp circular plasmid designated as pGR-1, with a GC content of 38.47 and 31.51 mol%, respectively. The coding genes, ribosomal RNAs, and transfer RNAs of the chromosome and plasmid pGR17 are listed in Table 2. Additional gene and function protein identification was performed using the KEGG database (Supplementary Table S1) and RAST (Supplementary Table S2). Based on the COG database of proteins (Tatusov et al., 2003), the proteins were divided into functional categories (Table 3). Specially, the immune-related proteins unique for bacteriocin-producing strains belonged to the defense mechanism category. The information of complete genome and COG is listed in Figure 1 and has been deposited at GenBank under the accession numbers CP033376 and CP033377.

**TABLE 2:** Features of *Enterococcus faecalis* Gr17 genome.

**Attributes**	**Chromosome**	**Plasmid**
Genome size (bp)	2,588,149	49,643
GC content (mol%)	38.47	31.51
rRNAs	18	0
tRNAs	67	0
Coding proteins	2,053	72

**TABLE 3:** COG categories of coding proteins in *Enterococcus faecalis* Gr17 genome.

**COG class**	**Name**	**Count**	**Proportion (%)**
C	Energy production and conversion	61	4.54
D	Cell cycle control, cell division, chromosome partitioning partitioning	16	1.19
E	Amino acid transport and metabolism	83	6.18
F	Nucleotide transport and metabolism	59	4.39
G	Carbohydrate transport and metabolism	179	13.33
H	Coenzyme transport and metabolism	27	2.01
I	Lipid transport and metabolism	40	2.98
J	Translation, ribosomal structure, and biogenesis	139	10.35
K	Transcription	96	7.15
L	Replication, recombination, and repair	100	7.45
M	Cell wall/membrane/envelope biogenesis	68	5.06
N	Cell motility	5	0.37
O	Posttranslational modification, protein turnover, chaperones	47	3.50
P	Inorganic ion transport and metabolism	80	5.96
Q	Secondary metabolites biosynthesis, transport, and catabolism	11	0.82
R	General function prediction only	112	8.34
S	Function unknown	147	10.95
T	Signal transduction mechanisms	32	2.38
U	Intracellular trafficking, secretion, and vesicular transport	12	0.89
V	Defense mechanisms	29	2.16

### Gene Cluster of Enterocin Gr17

**FIGURE 2:**
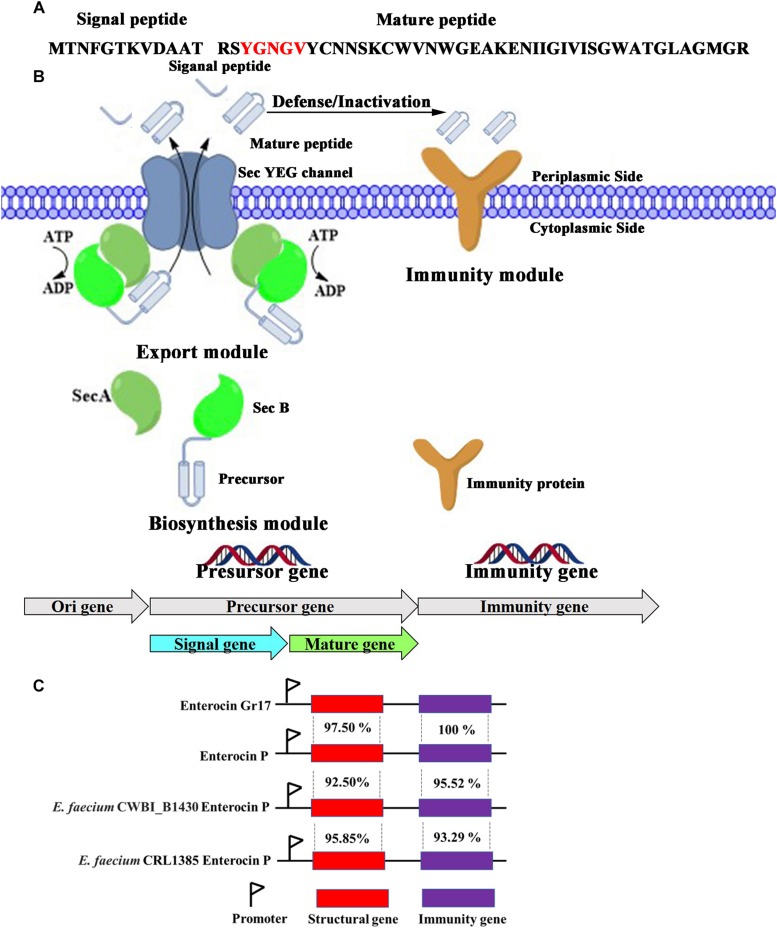
The amino acid sequences of precursor peptides encoded by structural gene **(A)**, biosynthetic mechanism of enterocin Gr17 in *E. faecalis* Gr17 **(B)**, and the comparison with other closely related enterocin gene clusters **(C)**.

The enterocin Gr17 gene was located on the plasmid. The conserved N-terminal YGNGV motif of mature class IIa bacteriocin was identified in enterocin Gr17 (Figure 2A). Thus, enterocin Gr17 was identified as a class IIa bacteriocin. Also, the type of N-terminal signal peptide of the enterocin Gr17 precursor revealed that the biosynthesis and secretion process of enterocin Gr17 were *via* the Sec-dependent secretion system. Generally, the biosynthetic gene cluster of enterocin Gr17 contained a structural gene, transporter genes, and immunity gene within three divergent operons (Beckwith, 2013). As shown in Figure 2B, the structural gene was composed of a precursor gene (EA467_13550), whose product could be cleaved to form an N-terminal leader peptide and mature antibacterial bacteriocin (Jack et al., 1995). The transporter genes encoded SecA protein (EA467_12775), an ATPase interacting with SecB protein and SecYEG complex; SecB protein (EA467_00140), which is responsible for recognition of nascent enterocin Gr17; and SecYEG complex (EA467_09045, EA467_06915, and EA467_12635), which facilitates the removal of signal peptide and secretion of mature enterocin Gr17. The immunity gene (EA467_13555) product could protect producer cells from mature enterocin Gr17. The comparison data with other closely related enterocin gene clusters are shown in Figure 2C. The structural gene displayed 97.50, 92.50, and 95.85% homology to enterocin P from *E. faecium* CWBI_B1430 and *E. faecium* CRL1385, respectively, and the immunity gene showed 100, 95.52, and 93.29% homology to the immunity genes of enterocin P from *E. faecium* CWBI_B1430 and *E. faecium* CRL1385, respectively.

### Virulence Factors and Antibiotic Resistance

The genes related to virulence factors, which included those encoding cytolysins (*cylA*, *cylB*, and *cylM*), gelatinase (*gelE*), sex pheromones (*cpd*, *cob*, and *ccf*), aggregation substance (*agg*), and extracellular surface proteins (*esp*, *efaAfs*, and *efaAfm*), were not found in the complete genome sequence of *E. faecalis* Gr17. Also, *E. faecalis* Gr17 did not contain antibiotic resistance genes encoding resistance to erythromycin (*ermB* and *ermC*), tetracycline (*tetM*, *tetS*, *tetO*, *tetK*, and *tetL*), ampicillin (*bla*), and vancomycin (*vanA*, *vanB*, and *vanC*). However, genes encoding resistance to ciprofloxacin (*gyrA* and *parC*) were located at EA467_08690 and EA467_03720.

### Purification of Enterocin Gr17

**FIGURE 3:**
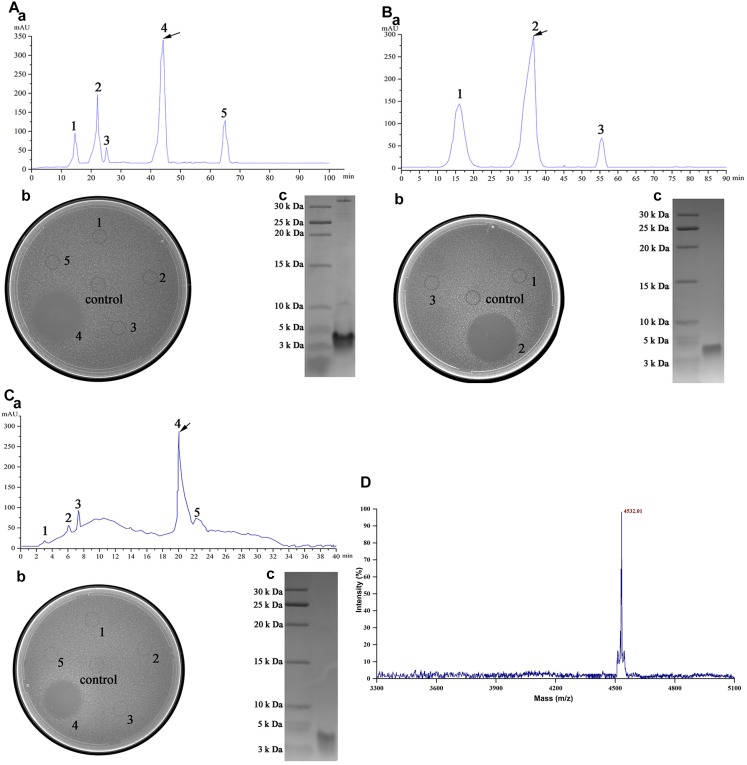
Purification of enterocin Gr17 from *E. faecalis* Gr17 by column chromatography. **(A)** Cation exchange column. **(B)** Gel filtration chromatography. **(C)** RP-HPLC. **(D)** Mass spectrum of enterocin Gr17 by MALDI–TOF–MS. a, process of purification; b, antibacterial activity of peaks against indicator strain by agar well diffusion assay; c, tricine–SDS–PAGE of activity fraction.

Crude enterocin Gr17 was extracted from fermentation supernatant by ammonium sulfate precipitation. Approximately 2.12-fold purification and 85.73% recovery were achieved (Table 4). SP-sepharose fast flow cation exchange column purification (Figure 3A) detected the active fraction at approximately 40 min elution time. The protein was purified 17.92-fold with 36.08% recovery. Sephadex G10 gel filtration chromatography purification yielded three different peptide fractions, with the active fraction eluted at approximately 30 min (Figure 3B). This process increased the antibacterial activity 65.6-fold, and 27.01% of the initial activity was recovered. RP-HPLC increased antibacterial activity 87.37-fold and the recovery was 5.77% (Figure 3C).

**TABLE 4:** Purification of enterocin Gr17.

**Purification Stage**	**Volume (mL)**	**Total protein (mg)**	**Total activity (AU)**	**Specific activity (AU/mg)**	**Purification fold**	**Recovery (%)**
Culture supernatant	1, 000	2,529	302, 100	119.45	1	100
Ammonium sulfate precipitation	30	1,021	259, 000	253.67	2.12	85.73
SP-sepharose fast flow	12	50.9	109, 000	2, 141.45	17.92	36.08
Sephadex G10	5	10.5	81, 600	7, 771.43	65.06	27.01
RP-HPLC	1	1.67	17, 430	10, 437.12	87.37	5.77

### Molecular Mass and Sequence of Enterocin Gr17

**FIGURE 4:**
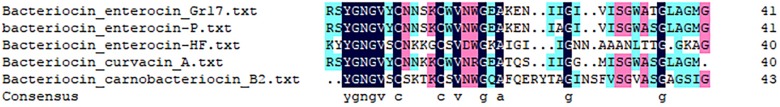
Alignment of reported class IIa bacteriocins. Alignments were obtained using DNAMAN 9.0 with default settings.

Matrix-assisted laser desorption/ionization time-of-flight mass spectrometry revealed that the molecular mass of enterocin Gr17 was 4,531.01 Da (Figure 3D). The complete genome sequence and molecular mass data indicated that the entire amino acid sequence was RSYGNGVYCNNSKCWVNWGEAKENIIGI VISGWATGLAGMGR. Due to the formation of an essential disulfide bond (Drider et al., 2006), the determined molecular mass was similar to the calculated mass. Enterocin Gr17 was different from reported bacteriocins in the protein BLAST search of the GenBank database^2^. Furthermore, sequence alignment with other mature class IIa bacteriocins showed that enterocin Gr17 has a novel amino acid sequence (Figure 4). The findings indicated that enterocin Gr17 from *E*. *faecalis* Gr17 is a novel class IIa bacteriocin with similarities to enterocin P.

### Antibacterial Spectrum of Enterocin Gr17

Enterocin Gr17 displayed strong antibacterial activity against Gram-positive bacteria (*L. monocytogenes*, *S. aureus*, *B. subtilis*, *B. cereus*, and *E. faecalis*) and poor antibacterial activity against Gram-negative bacteria (*E. coli*, *Salmonella enteritidis*, *Brochothrix thermosphacta*, *Pseudomonas aeruginosa*, *Pseudomonas fluorescens*, and *Cronobacter sakazakii*) and against the pathogenic yeast *Candida albicans* (Table 1).

### Stability of Enterocin Gr17

**FIGURE 5:**
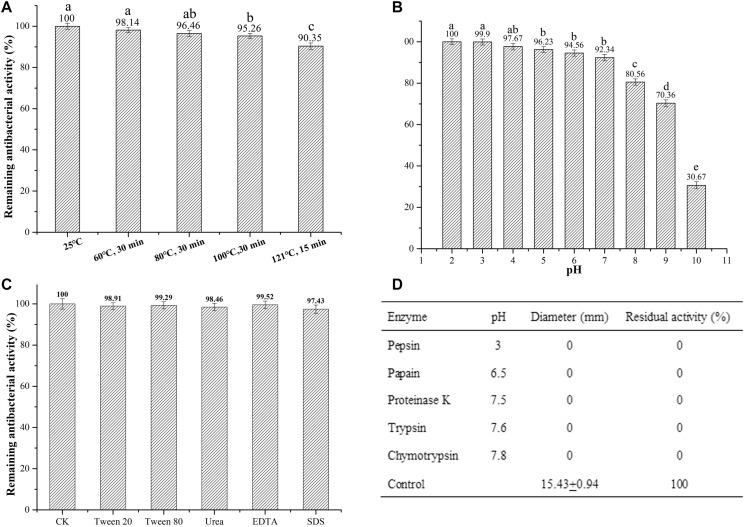
Effects of temperature **(A)**, pH **(B)**, surfactants **(C)**, and protease **(D)** on the stability of enterocin Gr17.

After heating at different temperatures, enterocin Gr17 still possessed antibacterial activity (Figure 5A). Enterocin Gr17 retained inhibitory activity at pH ranging from 2 to 10, but the activity was lost in pH 11 (Figure 5B). Surfactants did not decrease the activity (Figure 5C), and the activity was completely eliminated when incubated with proteolytic enzymes (Figure 5D).

## Discussion

Numerous enterocins from *E. faecium* have been reported. These include the class IIa enterocin NKR-5-3C from *E. faecium* NKR-5-3C (Himeno et al., 2012), class IIa enterocin TW21 from *E. faecium* D081821 (Chang et al., 2013), class IIc enterocin AS-48 (Abengózar et al., 2017), and class IId enterocin K1 (Ovchinnikov et al., 2017). Even though features of the various enterocins have been determined, genome information and details of the biosynthesis mechanisms have been unknown. Also, the genome information of enterocin-producing strains, including *E. faecium* ICIS 96 (Pashkova et al., 2018), *E*. *faecium* CRL1879 (Bonacina et al., 2017), and *E*. *faecium* M3K31 (Arbulu et al., 2016), has been identified, but the features and biosynthesis mechanisms have also been unclear. The present findings reveal the biosynthesis mechanism and features of the novel class IIa enterocin Gr17 in the novel strain *E*. *faecalis* Gr17.

The potential of bacteriocins and bacteriocin-producing LAB as biopreservatives for fermented fish product has inspired searches for strains with potent antagonistic effects against food-poisoning microorganisms from fermented fish. Recent studies have isolated and screened bacteriocin-producing strains from fermented fish in different countries (Table 5). However, bacteriocin-producing strains isolated from Chinese fermented fish have not been adequately studied; as far as we know, the only bacteriocin-producing bacterium is *Lactobacillus plantarum* LPL-1, which was isolated from Chinese fermented sturgeon fish products (Wang et al., 2018). In this manuscript, we isolated a novel bacteriocin producer, *E. faecalis* Gr17, from Suan yu, a Chinese traditional low-salt fermented whole-fish product. This is the first report of a novel bacteriocin-producing strain from Suan yu. The antibacterial activity and spectrum of bacteriocins are important factors for the application of bacteriocins in the food industry. The novel amino acid sequence of bacteriocins could provide a foundation for exploring the relationship between bacteriocin structure and antibacterial activity. The characteristics, molecular mass, and amino acid sequence of enterocin Gr17 are obviously different from some well-known enterocins.

**TABLE 5:** The bacteriocin-producing strains from fermented fish of different countries.

**Bacteriocin-producing strain**	**Bacteriocin**	**Source**	**References**
*Weissella cibaria* 110	Weissellicin 110	Thai fermented fish product (plaa-som)	Srionnual et al., 2007
*Staphylococcus hominis* KQU-131	Nukacin KQU-131	Thai fermented fish product (Pla-ra)	Wilaipun et al., 2008
*Pediococcus pentosaceus* CFF4	Bacteriocin CFF4	Cambodian fermented fish (pha ak trey)	Peng et al., 2017
*Lactobacillus plantarum* LPL-1	Plantaricin LPL-1	Chinese fermented sturgeon fish products	Wang et al., 2018
*Lactococcus lactis* ssp. *Lactis* HKBT-9	Bacteriocin HKBT-9	India fermented Fish Product (Hukuti Maas)	Kumar et al., 2014
*Weissella confusa* N23	Bacteriocin N23	Thai fermented meat and fish products	Pringsulaka et al., 2012
*Lactococcus lactis* NK24	Lacticin NK24	Korean fermented fish product (jeot-gal)	Lee and Paik, 2001
*Enterococcus faecium* DB1	Enterocin DB1	Korean fermented fish product (gajami sikhae)	Lee and Kim, 2010
*Enterococcus faecalis* F4-9	Enterocin F4-9	Egyptian salted-fermented fish	Maky et al., 2015
*Enterococcus faecium* NKR-5-3	Enterocin NKR-5-3	Thai fermented fish products	Himeno et al., 2012

Cytolysin encoded by *cylA*, *cylB*, and *cylM* is a bacterial toxin that increases the risks of illness and death. Gelatinase encoded by *gelE* participates in the initiation and propagation of inflammatory processes. Sex pheromones encoded by *cpd*, *cob*, and *ccf*, and aggregation substance encoded by *agg* cause cell aggregation and facilitate the transfer of virulence factors and antibiotic resistance genes. Extracellular surface proteins encoded by *esp*, *efaAfs*, and *efaAfm* can promote adhesion of cells and protect the cells from the immune system. These virulence factors were not detected in *E. faecalis* Gr17, which suggests that the strain is non-virulent and relatively safe for consumers. Also, *E. faecalis* Gr17 did not contain genes encoding resistance to erythromycin, tetracycline, ampicillin, and vancomycin, but could potentially be resistant to ciprofloxacin. We screened *E. faecalis* Gr17 from the fermented whole-fish product Suan yu. Ciprofloxacin was often used to protect fish from pathogenic bacteria in aquaculture. It is conceivable that *E. faecalis* Gr17 may be transmissible from fish by highly efficient gene transfer mechanisms. Still, the available genotypic evidence of potential virulence factors and antibiotic resistance indicates that the strain may be safe to use.

Class IIa enterocins can be exported from cells by the Sec system (e.g., enterocin P in *E. faecium* P13; Cintas et al., 1997) and the ABC transporter (e.g., enterocin B in *E. faecium* T136; Casaus et al., 1997). Also, the biosynthetic mechanisms of other enterocins, which are termed leaderless bacteriocins, are unclear; these enterocins include enterocin K1 (Ovchinnikov et al., 2017) and EntEJ97 (Ovchinnikov et al., 2014). The biosynthetic mechanism of enterocin Gr17 belonged to the Sec system according to the complete genome information of *E. faecalis* Gr17. The genes relevant to the structural gene, transporter genes, and immune-related gene were identified. Moreover, the specific function of relevant genes was predicted by bioinformatic analysis. This genome information of *E. faecalis* Gr17 provides a better understanding of the biosynthesis mechanism of enterocin Gr17. Further investigations of the relevance between the quorum sensing system and biosynthesis of enterocin Gr17 will be carried out.

The precursor of class IIa enterocins is composed of signal peptide and mature peptide (Jack et al., 1995). Generally, the cleavage site between the signal peptide and mature peptide could be determined by bioinformatic analysis and MALDI–TOF–MS. According to the genome information, the amino acid sequence of the precursor was determined. Enterocin Gr17 was identified as a class IIa bacteriocin because of the N-terminal conserved YGNGV motif of mature class IIa bacteriocin. To determine the molecular mass of the mature peptide, enterocin Gr17 was purified by salt precipitation, cation exchange, gel filtration chromatography, and RP-HPLC. Different from other enterocins, such as enterocin RM6 (7145.0823 Da) (Huang et al., 2013), enterocin TW21 (5302.98 Da) (Chang et al., 2013), and enterocin AS-48 (7149 Da) (Abengózar et al., 2017), the molecular mass of purified enterocin Gr17 was 4,531.01 Da, which corresponded to a calculated molecular mass of 4,533.11 Da due to the formation of a disulfide bond. To some extent, the molecular mass result confirmed the novelty of enterocin Gr17. Especially, the results of genome information and MALDI–TOF–MS revealed that the amino acid sequence of mature enterocin Gr17 was RSY GNGVYCNNSKCWVNWGEAKENIIGIVISGWATGLAGMGR. A BLAST search of the NCBI database^3^ for mature enterocin Gr17 revealed its difference from reported class IIa enterocins. Therefore, enterocin Gr17 was confirmed as a novel class IIa enterocin. Compared with known class IIa bacteriocins, the enterocin Gr17 possesses a similar N-terminal sequence of xxYGNGVxC.

The antibacterial activity of enterocins can provide a basis for their application as food biopreservatives. In contrast to the narrow spectrum bacteriocins enterocin W (Sawa et al., 2012), enterocin A (Ennahar and Deschamps, 2000), enterocin 416K1 (Sabia et al., 2002), and enterocin CRL35 (Salvucci et al., 2007), enterocin Gr17 possessed antibacterial activity against *L*. *monocytogenes*, *S*. *aureus*, *B*. *subtilis*, *B*. *cereus*, *B. anthracis*, *E*. *coli*, *S*. *enteritidis*, *P*. *aeruginosa*, *P. fluorescens*, *E. faecalis*, *E*. *sakazakii*, and *C*. *albicans* (Table 1). These food-borne pathogenic and spoilage bacteria are also most frequently detected in the food industry and in medical science. Numerous authors also combined bacteriocins with the hurdle technology to inhibit food-borne pathogenic bacteria (Leistner, 2000), such as chemical chelators (sodium tripolyphosphate and EDTA) and physical methods (pH, temperature, high hydrostatic pressure, and pulsed electric field) (Thomas et al., 1998; Ananou et al., 2005; Martínez et al., 2008; Khan et al., 2015; Prudêncio et al., 2015). Thus, enterocin Gr17 has the potential to be applied with the hurdle technology to control food quality. The antibacterial activity of enterocin Gr17 makes it a good candidate for the preservation of various types of foods.

Concerning the application of enterocin Gr17 in the biopreservation of foods, its stability during different chemical treatments is essential. Enterocin Gr17 was very stable to a wide range of pH, high temperatures, and surfactants. These features are typical of the numerous bacteriocins that have been characterized, such as enterocin from *E*. *faecium* JCM 5804T (Park et al., 2003), enterocin ON-157 from *E*. *faecium* NIAI 157 (Ohmomo et al., 2000), and enterocin RM6 (Huang et al., 2013). The thermal stability of enterocin Gr17 indicates that it is valuable for use in dairy products and heat-processed foods. The pH stability could allow its use with slightly alkaline, neutral, and acidic foods. The surfactant stability could be ideal for use in emulsified foods. The antibacterial activity of enterocin could be destroyed by human digestive enzymes. To a certain extent, enterocin Gr17 could be safely used in the food industry, and safety for the human health remains to be confirmed by toxicity experiments in the future. Therefore, enterocin Gr17 is a good candidate as a safe biopreservative in the food industry.

Generally, the formation of pores in the membrane of cells is lethal. To definitively analyze the antibacterial mechanism of enterocin Gr17 against food-borne pathogenic and spoilage bacteria, further investigations on morphology changes will be done using scanning tunneling microscopy, transmission electron microscopy, and atomic force microscopy, and the exchange of molecules between inner and outer membrane of Gram-negative bacteria will be explored using proton motive force. As well, ATP and inorganic ions will be examined. Metabolomics, transcriptomics, and proteomics data will be combined to clarify the antibacterial mechanism.

## Conclusion

Bioinformatic analysis clarified the biosynthetic mechanism of enterocin Gr17 in the novel strain *E. faecalis* Gr17. Based on bioinformation and MALDI–TOF–MS, enterocin Gr17 from *E*. *faecalis* Gr17 was identified. The entire amino acid sequence was determined to be RSYGNGVYCNNSK CWVNWGEAKENIIGIVISGWATGLAGMGR. Enterocin Gr17 exhibited bactericidal activity, sensitivity to enzymes, and stability to chemicals, elevated temperature, and pH. Therefore, enterocin Gr17 is a promising stable and safe biopreservative in various types of foods. Future investigations on the bactericidal mechanism of enterocin Gr17 will be carried out.

## Author Contributions

YW, GL, and CW designed the experiments. YW, XL, XH, and DX performed the experiments. YW, GL, YZ, and AM analyzed the results and wrote the manuscript.

## Conflict of Interest Statement

The authors declare that the research was conducted in the absence of any commercial or financial relationships that could be construed as a potential conflict of interest.

## Footnotes

1 http://www.cbcb.umd.edu/software/glimmer/

2 www.ncbi.nlm.nih.gov/BLAST

3 www.ncbi.nlm.nih.gov/BLAST

## Supplementary Material

TABLE S1 Additional gene and function protein identification using the KEGG database.

TABLE S2 Additional gene and function protein identification by RAST.
